# A refinement to the formalin test in mice

**DOI:** 10.12688/f1000research.18338.2

**Published:** 2019-08-02

**Authors:** Douglas M Lopes, Heather L Cater, Matthew Thakur, Sara Wells, Stephen B McMahon

**Affiliations:** 1 Neurorestoration Group, Wolfson Centre for Age-Related Diseases, King's College London SE1 1UL, London, UK; 2 MRC Harwell Institute, Harwell Campus, Didcot, Oxfordshire, OX11 0RD, UK

**Keywords:** Pain, Formalin, Nociception, Behaviour, Refinement, 3Rs

## Abstract

The constant refinement of tests used in animal research is crucial for the scientific community. This is particularly true for the field of pain research, where ethical standards are notably sensitive. The formalin test is widely used in pain research and some of its mechanisms resemble those underlying clinical pain in humans. Immediately upon injection, formalin triggers two waves (an early and a late phase) of strong, nociceptive behaviour, characterised by licking, biting, lifting and shaking the injected paw of the animal. Although well characterised at the behaviour level, since its proposal over four decades ago, there has not been any significant refinement to the formalin test, especially those combining minimisation of animal distress and preservation of behavioural outcomes of the test.  Here, we propose a modified and improved method for the formalin test. We show that anaesthetising the animal with the inhalable anaesthetic sevoflurane at the time of the injection can produce reliable, robust and reproducible results whilst animal distress during the initial phase is reduced. Importantly, our results were validated by pharmacological suppression of the behaviour during the late phase of the test with gabapentin, the anaesthetic showing no interference with the drug. In addition, we demonstrate that this is also a useful method to screen for changes in pain behaviour in response to formalin in transgenic lines.

## Introduction

Since first reported over 40 years ago, the formalin test ^1^ has been widely used in pain research and is known to capture mechanisms that are likely to be relevant to many pain patients in clinic ^2, 3^, including the poorly localized, burning and throbbing pain sensation ^4^. The unique feature of the formalin test is that it triggers two phases of nociceptive behaviour: the first one is directly linked to the stimulation of primary sensory neurons and is followed by a second phase, which is associated with inflammation and involves central sensitisation ^1, 5– 7^. These phases are marked by striking pain behaviour in which the animals lick, bite, lift and shake the injected paw. Over many years, different groups have extensively characterised the test, demonstrating robust and reproducible quantitative behaviour outcomes ^5, 8– 12^.

The constant refinement of experimental procedures involving animals is important for the whole research community but is particularly important for the field of pain research, due to the obvious ethical implications of this type of research. Regulations regarding the use of animals for research in the UK dates back to the 19th century, with strict safeguards to avoid or minimise animal suffering and cruelty, as well as ensuring high animal welfare standards are met ^13, 14^. Furthermore, the 3Rs concept (reduce, refine and replace) ensures that for every experiment, the use of animals is absolutely necessary ^13, 15– 17^. Further to these ethical considerations, and of particular relevance to the formalin test, other evidence suggests that restraining also induces stress, behavioural and other physiological changes in the animals ^18– 25^, including hyperalgesia ^26– 28^, which can impact the outcome of the test. As highlighted in the above guidelines and given the current standard procedure for the formalin test, where the animals are physically restrained and may experience unnecessary levels of stress, the present study aimed to refine the current method used for the injection of formalin, without compromising its reproducibility. We posed the question of whether anaesthetising the animal at the time of formalin injection could result in more consistent injections and reduce the stress experienced by the animals, without losing the behavioural effects that formalin triggers.

We show that the use of an inhalable anaesthetic during the time of formalin administration minimises animal stress and improves injection consistency. Whilst the anaesthetic reduced the behaviours observed during the first response phase, it appears not to affect the responses observed during the second phase of the test. We validated our proposed method by showing its sensitivity to a known analgesic agent, gabapentin, as well as its efficacy in different transgenic mouse lines. Together, our data present a refined method for the formalin test, whilst also demonstrating that the second phase can occur without a behavioural response during the first phase.

## Methods

### Ethical statement

All experiments were performed in accordance with the UK Animals (Scientific Procedures) Act 1986 and Local Ethical Committee approval. All efforts were made to minimise the suffering of animals during the experiments by carefully following the procedures.

### Experimental animals

In all experiments, adult (11 to 13 weeks of age) homozygous and wildtype, male and female littermates were used. For the gabapentin and sevoflurane experiments, wildtype C57BL/6NTac mice were used. Studies on the anaesthetised groups were performed on animals bred at Mary Lyon Centre (MLC; Harwell, UK), whereas the remaining animals were bred at King’s College London (KCL; UK). For the experiments using transgenic animals, mice carrying the null alleles *Pink1* ^tm1b(EUCOMM)Wtsi^ and *Slit1* ^tm1b(Komp)Wtsi^ were generated at Harwell (UK), as part of the International Mouse Phenotyping Consortium (IMPC) and maintained as heterozygotes on a C57BL/6NTac background. The colony was intercrossed and genotyped by an independent experimenter, to ensure effective blinding during behavioural testing. Males and females were used in the experiments (except for the Gabapentin study, Figure 3). No sex-difference in behavioural responses to the formalin test was found in this study. Details on both the visual representations and statistical analysis demonstrating no differences between the genders are presented on Supplementary Figure 1. The transgenic lines chosen in this study were part of a parallel neuroscience program study being carried out at the MLC. In addition, it has been suggested a link between Pink1 and nociceptive processing ^29, 30^ and Slit1 expression and peripheral injury ^31– 36^.

### Housing and husbandry

 Animals were housed in IVC cages (Tecniplast – 1284L and 1285L, with autoclaved Datesand Aspen bedding) in groups of 2 to 5 per cage, under 12-hour-on/12-hour-off cyclic lighting (30minutes dusk to dawn, dawn to dusk period), at controlled temperature (21 ± 2°C) and humidity (55 ± 10%) conditions. Cage bases were changed weekly. The mice had free access to filtered water (25 p.p.m. chloride) and were fed *ad libitum* on a commercial diet - either Rat and Mouse Diet No. 3 [RM3] (Special Diet Services, Essex, UK), composed of 5.3% fat [corn oil], 21.2% protein, 57.4% carbohydrate and 4.6% fibre (provided at the MLC; Harwell BSU facility), or PicoLab Rodent Diet 5053 (LabDiet St. Louis, MO, USA), composed of 21% protein, 4.5% fat and 6% fibre (provided at King’s College London BSU facility). All mice housed in the same facility received the same food. Food was irradiated to 2.5 Mrads. All mice went through daily health checks for general physical and health appearance (e.g. coat, eyes and ears appearances, fighting wounds), bedding/water bottle appearance and any signs of distress. The MLC and KCL are specific pathogen free centres.

### Sample size and allocation to experimental groups

A total of 95 mice were used for the experiments: Sevoflurane experiments: N = 16 anaesthetised and N = 8 non-anaesthetised; Gabapentin experiments: N = 20 gabapentin and N = 16 controls; Pink1 experiments: N = 8 Pink1 -/- and N = 5 controls and Slit1 experiments :N = 11 Slit1 -/- and N = 11 controls. Animals were allocated using simple random randomization, i.e. subjects to each group purely randomly for every assignment.

### Experimental procedure

**Figure 1.**
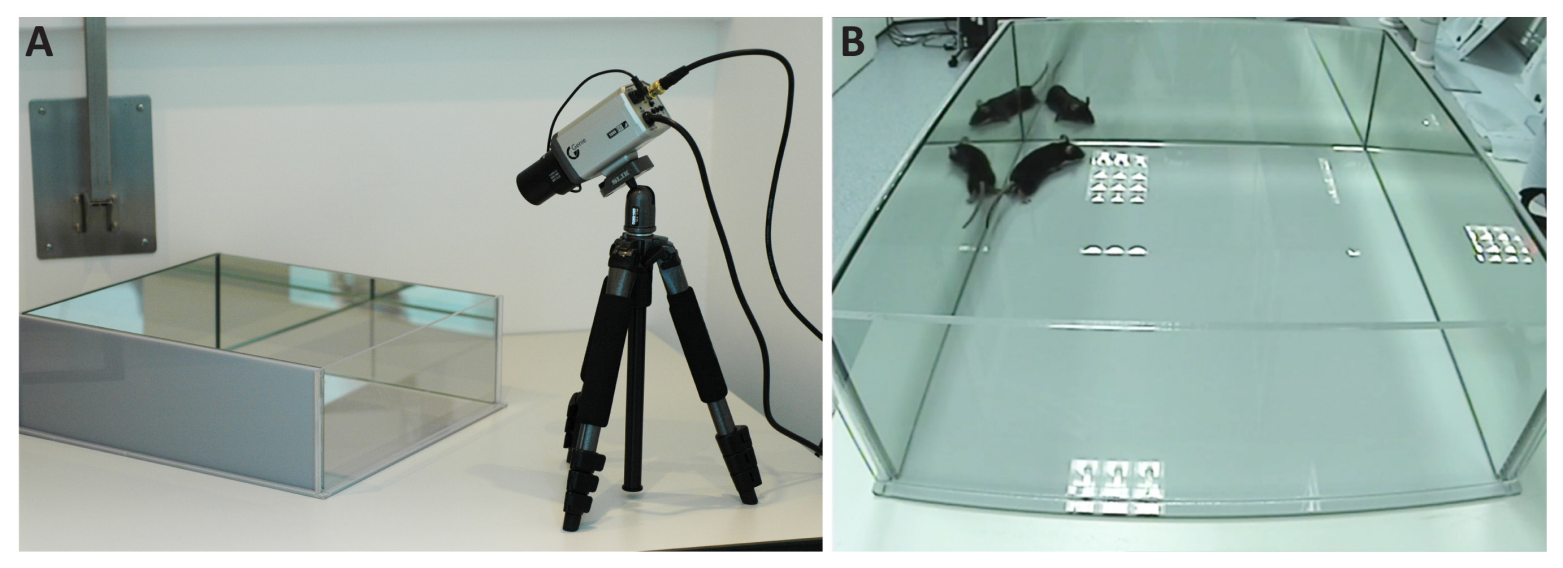
Formalin test apparatus. ( **a**) Perspex acrylic glass arenas (36 x 40 x 13 cm), consisting of three mirrored and one transparent wall, were used for the formalin test. The recording camera was placed facing the arena, at an approximate 30° angle to the arena surface and 50 cm away from the transparent wall, so all mouse behaviour could be easily recorded for later analysis. ( **b**) Video still of an experiment, showing that the behaviour of the animal could be meticulously monitored and accessed.

Mice were anaesthetised by inhalation of sevoflurane (5% flow) (Zoetis, UK) for 2 minutes, followed by subcutaneous injection of formalin (20ul at 1.85% concentration) (Sigma, UK, Cat Nº 252549) into the right hind paw. A 30-gauge needle was used to perform the injections. A single animal was then placed into the arena (details below, and in Figure 1) and camera recording was started. For the gabapentin experiments (experiments related to Figure 3), gabapentin (Sigma, UK, Cat. № G154) was injected intraperitoneally at a concentration of 50mg/kg, immediately prior to the injection of formalin. All experiments were performed during the light cycle (usual starting time 9am).

Perspex acrylic glass arenas (36 x 40 x 13 cm), consisting of three mirrored and one transparent wall, were built in house (please refer to the apparatus set up in Figure 1). A recording camera was positioned to face the arena, at an approximate 30° angle to the surface on which the arena is placed ** and 50 cm away from the transparent wall, so all mouse behaviour could be easily recorded for later analysis. The set-up also allows annotations to be made by independent experimenters to provide accurate and reproducible observations of the changes in behaviour. Pain behaviour was scored using a stopwatch when flinching, licking and flinching continued by licking, flicking or shaking of the paw were initiated and timer was stopped once behaviour ceased. Limping, altered locomotion or grooming of other parts of the body were not counted as pain behaviours. The first phase of the test was designated as the time between zero and 15 minutes after the formalin injection, whereas the late (or second) phase was from 15 minutes onwards (up to 45 minutes). Animals were humanely culled using Schedule 1 at the end of the test. All experiments were annotated by an experimenter, blinded to the genotype and treatment.

### Statistical analysis

Statistical analyses were performed using OriginLab 2017 software (Origin Group Corp.) (for area under the curve) and SPSS Statistics 20 (ANOVA repeated measures). For all sets of samples, normality tests were performed using the Shapiro-Wilk test, to check whether the data fitted in a Gaussian distribution (95% confidence intervals). Power calculations were performed using the Columbia University Biomath Calculator, following the guidelines previously described ^37^. For details on the power calculation for each individual experiment, please refer to the *Extended data* section of this manuscript ^38^. For all hypothesis testing, the minimum level of statistical significance adopted (p value) was at 0.05 - where if there was a 5% or less chance (5 in 100 or less) against the null hypothesis, so the latter would be rejected. The AUC was calculated in relation to the pain response (sec) over time.

## Results

**Figure 2.**
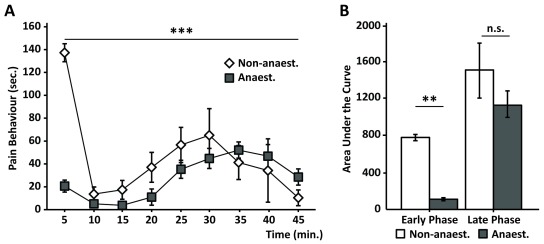
Sevoflurane during formalin injection only impacts on the first phase of the test. ( **a**) Quantification of pain response over time of animals which were anaesthetised during the formalin injection (grey squares) and non-anaesthetised animals (white diamonds) shows distinct behaviors in the early phase of the test (0 - 15 min. after injection) but similar behaviours in the later phase (15 minutes onwards). ANOVA repeated measures: time: F _(4,80)_: 5.92; p<0.001; time*group: F _(1,34)_: 5.357; p =0.001. ( **b**) Plots representing the area under the curve show a significant reduction in pain response in anaesthetised vs. non-anaesthetised animals during the early phase of the test, but not during the late phase (p < 0.01; One-way ANOVA; N = 16 anaesthetised and N = 8 non-anaesthetised). Graphs represent means +/- SEM.

The formalin test is a robust model to study pain, and it has been demonstrated to be sensitive to various analgesic drugs ^10, 39– 42^. The stress and anxiety-like behaviours triggered by aversive handling and restraining of the animal whilst it is being injected with the formalin, together with exposure to an unfamiliar environment, may interfere with the stimulus and the outcome of the test ^43^. To check whether restraining stress could be minimised during the formalin injection, without having a major effect on the results of the test, we anaesthetised the animals with sevoflurane at the time of injection. Our results show that the anaesthetic virtually abolished the first phase of the test, whilst still preserving the second phase ( Figure 2a) ^38^. When compared to non-anaesthetised mice, we observed a reduction in pain behaviour of approximately 90% ( Figure 2b) for anaesthetised mice during the first 15 minutes of the test (first phase). Notably, no significant changes in behaviour following the injection of anaesthetised mice were observed during the late phase of the test (20 – 45 min after injection) when compared to the non-anaesthetised group ( Figure 2a & 2b).

**Figure 3.**
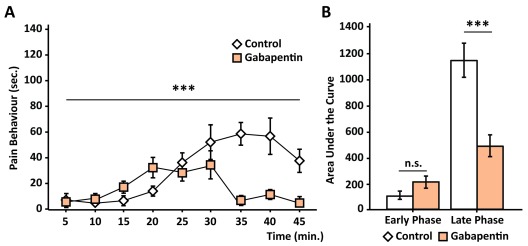
Use of anaesthetic does not interfere with gabapentin treatment. ( **a**) Graph representing behavioural response to formalin injection over time. Anaesthetised animals which receive gabapentin (peach squares) present a diminished response to formalin injection in comparison to the control group (white diamonds; anaesthetic only). ANOVA repeated measures, time: F _(3.8, 129.6)_: 7.998; p< 0.001; time*group: F _(3.8, 129.634)_: 6.92; p <0.001. ( **b**) Bar graphs representing the area under the curve show no difference between the groups in the early phase of the test, but a clear effect of the drug during the late phase (p < 0.001; One-way ANOVA; N = 20 gabapentin and N = 16 control). Graphs represent means +/- SEM.

The effect of the anti-epileptic drug gabapentin as an analgesic is well documented. Indeed, many studies demonstrate that gabapentin attenuates nociceptive behaviour following formalin injection, specifically during the late phase of the test ^44– 49^. As the formalin test is widely used as a powerful tool to screen the analgesic effect of novel compounds at the preclinical level, we next tested whether gabapentin would also decrease the nociceptive behaviour observed after formalin injection using our newly proposed method. Our data demonstrate that in the gabapentin-treated group, there was a clear decrease in pain behaviour in the late phase of the test when compared to the control group ( Figure 3a). Gabapentin treatment led to a reduction of over 50% in nociceptive behaviour after formalin injection ( Figure 3b) in comparison with the non-treated group.

The use of transgenic animals in research has been crucial in elucidating numerous biological mechanisms involved in both health and disease, including in the field of pain research. Given the importance of transgenic mice and the results of behavioural tests to screen potential molecular targets, we went on to investigate whether our refined formalin method could be effective for newly generated transgenic mouse lines. For these experiments, we used two knockout mouse lines in which the target genes are known to be expressed in the dorsal root ganglia (DRG) ^50– 52^ and in spinal cord neurons ^53^.

**Figure 4.**
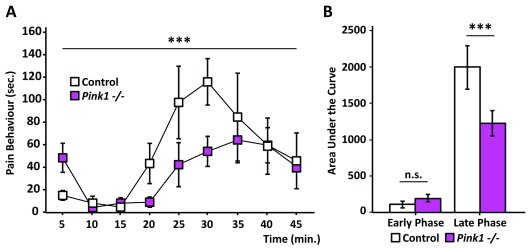
PTEN-induced kinase 1 knockouts ( *Pink1* -/-) present a distinct behaviour in the refined formalin test. ( **a**) Graph showing the behavioural response of *Pink1* -/- (pink squares) and their control littermates (white squares) over time. *Pink1* -/- nociceptive behaviour in the late phase is reduced in comparison to the control group. ANOVA repeated measures, time: F _(3.8, 41.5)_: 6.772; p< 0.001. ( **b**) Area under the curve plot shows that whilst both groups have very similar response to formalin in the early phase of the test, *Pink1* -/- mice display a lower pain response to formalin in the late phase (p < 0.001; One way ANOVA; N = 8 *Pink1* -/- and N = 5 control). Graphs represent means +/- SEM.

The PTEN-induced kinase 1 ( *Pink1*) gene has been extensively studied in the context of Parkinson’s disease ^54, 55^ and has been also linked to nociceptive processing ^29, 30^. Our data show that animals lacking *Pink1* have a significantly lower response to the formalin test ( Figure 4a). Although the *Pink1* knockout group appeared to be marginally more responsive during the first 5 minutes of the test, their overall response to formalin injection during the first phase was very similar to their control littermates ( Figure 4b). In contrast, their nociceptive behaviour during the late phase was reduced by 40% in comparison to the behaviour of the control group ( Figure 4b). It appears that, despite being less responsive to the formalin, the *Pink1* knockout animals have a steadier response in the second phase when compared to the control littermates, with no obvious peak in the response over time ( Figure 4a).

**Figure 5.**
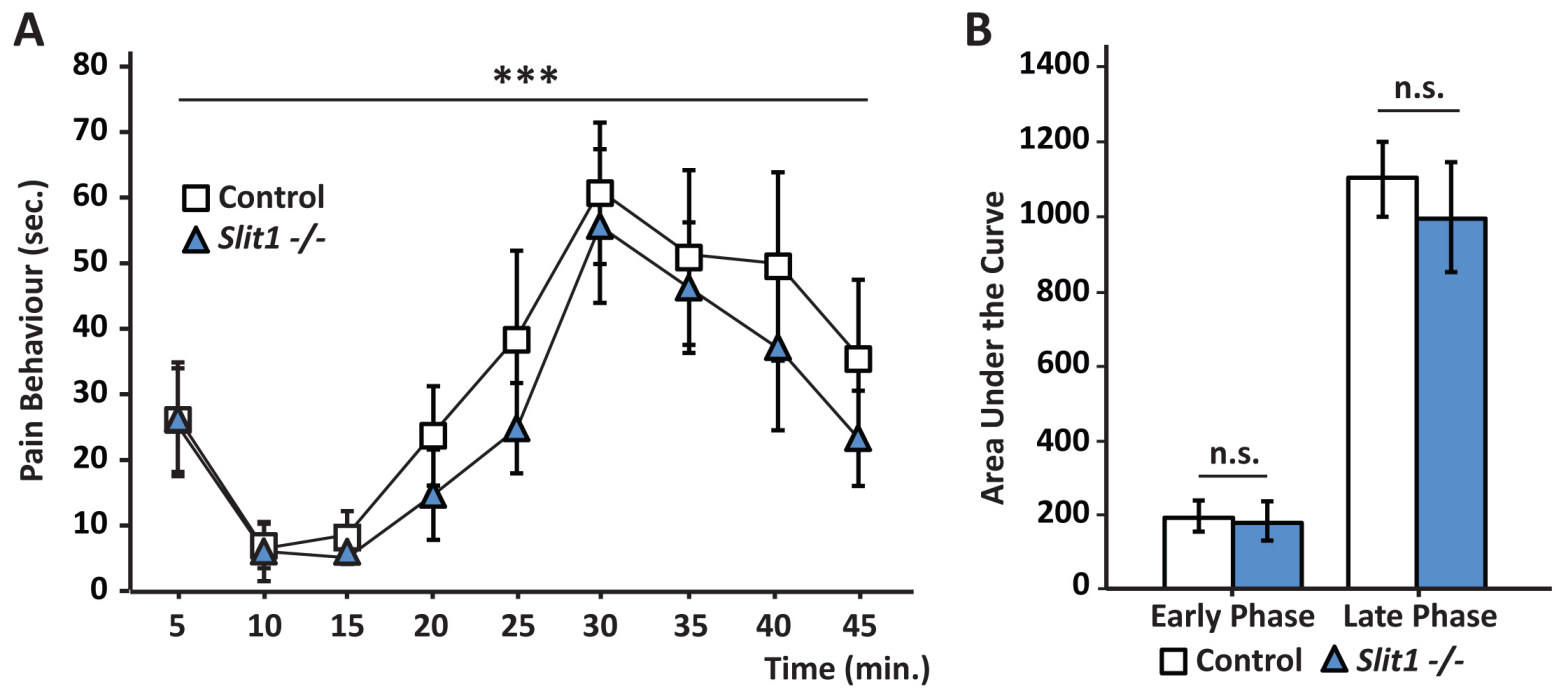
*Slit1* knockout mice behave similarly to their control littermates in the refined formalin test. ( **a**) Graphs depicting the behavioural response to formalin injection in *Slit1* -/- (blue triangles) mice and control littermates (white squares). Both groups show similar pain behaviour in both phases of the formalin test. ANOVA repeated measures, time: F _(3.6, 72.9)_: 0.928; p< 0.001. ( **b**) Area under the curve graph further shows the similarities in pain behaviour between *Slit1* -/- and control groups. (n.s., where p> 0.05; One-way ANOVA; N = 11 *Slit1* -/- and N = 11 control). Graphs represent means +/- SEM.

Following the same principle, we went on to screen a transgenic mouse line with a disrupted Slit Guidance Ligand 1 ( *Slit1*) gene, using our refined formalin test. Slit1 is a secreted protein, which has been reported to be involved in DRG and spinal cord development, and previous studies have suggested a link between injury and increased *Slit1* expression ^31– 36^. Our data show no overall difference in pain behaviour in the formalin test between the group with disrupted *Slit1* function and their control littermates ( Figure 5a). As with control mice, the pain behaviour during the first phase of the test is dramatically reduced due to the anaesthetic. Notably, a loss of function of the *Slit1* gene did not lead to any change in the nociceptive behaviour during the late phase of the test ( Figure 5a and 5b), as both groups spent a similar amount of time exhibiting pain behaviour ( Figure 5b).

## Discussion

In this study we proposed a modified and improved method for the formalin test. We showed that by anaesthetising the animal at the time of the injection reliable, robust and reproducible results are produced, whilst diminishing stress to the animals. Our newly proposed method showed that whilst the pain behaviour in the first phase was suppressed by the anaesthetic, the response in the late phase remained comparable to the control, non-anaesthetised group. Our results were validated by pharmacological suppression of the late phase with gabapentin. Furthermore, we demonstrate that this is a useful method to use while screening transgenic lines for changes in pain behaviour in response to formalin.

Reducing the levels of stress in the animals is of great advantage when performing behaviour tests. Many studies have shown that mishandling of laboratory animals can have profound impacts on some behavioural tests ^43, 56, 57^. In particular, animal restraining has a drastic, negative effect on anxiety levels ^58, 59^, with evidence suggesting that the adverse effects also extend to pain behaviour, where it can lead to areflexia, hyperalgesia and, in some cases, abnormal flinching ^60– 64^. Given the potential adverse effect that stress, resulting from either mishandling or restraining the animal during the administration of a drug or compounds, can have on pain behaviour, we propose the use of anaesthesia during the drug application. In our method, we show that animals can be injected with formalin without causing any apparent distress, whilst increasing the reproducibility of the test, as consistency in the site of injection as well as the volume injected is improved under anaesthesia. Indeed, due to ethical considerations, it has recently been suggested that brief anaesthesia immediately before injection would be beneficial for the animals, as well as increasing the accuracy of substance application, considering that the site of formalin injection is crucial ^65^. Importantly, we show that the outcome of the test is similar to that of the traditional method and that, despite the anaesthesia reducing the pain behaviour during first phase, the second phase behaviour times remained almost identical. Notably, our method has a significant advantage over a previous study which employed different inhalation anaesthetics, shown to have a negative impact on the early and late stages of the test ^66^, suppressing the pain behaviour in both phases dramatically and being remarkably different to non-anaesthetised animals. In summary, our method resulted in minor changes to overall behaviour responses and provided significant advantages for ethical and stress-free animal handling.

Central sensitisation by formalin appears to be the most crucial aspect of the test when evaluating nocifensive behaviour. The underlying mechanisms of the formalin test are not fully understood. Historical experimental data indicate that the behavioural response observed after the injection is solely due to the direct stimulation and activity of C-fibre nociceptors ^1, 9, 67^, whereas subsequent studies suggest the involvement of Aδ and non-nociceptive Aβ-fibres ^68, 69^. Whilst there is still controversy regarding the circuitry and cellular and molecular mechanisms triggered by formalin, subjects which are beyond the scope of this study, we demonstrate that the second phase of the test can be used to screen pain behaviour independently of the first phase. Supporting our findings, studies showed that knockout or ablation of distinct nerve fibre populations in animal models resulted mostly in a reduction of the pain behaviour in the second phase of the test, while the first phase was not necessarily affected ^69– 71^. Furthermore, these studies show that, in all instances in which the first phase is affected, the second phase will also be influenced ^69– 71^, demonstrating therefore that suppressing or diminishing the pain behaviour during the first phase of the test is almost inconsequential when screening phenotypes and testing drugs. Therefore, our study highlights that the formalin test could be improved by diminishing unnecessary animal distress without compromising the results, given that the first phase of the test is, in most cases, not very informative.

The second phase of the formalin test alone can be used for phenotypic screening. We confirmed the sensitivity of our modified protocol by showing that the effects of the extensively characterised analgesic, gabapentin ^44– 48^ are maintained as expected. We further validated the sensitivity of the modified formalin procedure in two different transgenic lines. The mitochondrial kinase *Pink1* has been extensively studied, and mutations in this gene are notoriously linked to neuronal dysfunction in Parkinson’s disease ^55, 72– 74^. Previous studies also linked the loss of function of *Pink1* in humans with abnormal pain sensation, where subjects present a higher mechanical and pressure threshold ^29, 30^. Notably, and similarly to the phenotype found in humans, we show here that animals with a disrupted *Pink1* gene display lower nociceptive behaviour after formalin injection. However, it cannot be excluded, the hypoalgesic phenotype can be due to symptoms arising from Parkinson’s disease itself - as *Pink1*-null mouse models might present motor dysfunction ^75^. Therefore, our results not only present for the first time a pain phenotype in a *Pink1*-null rodent model in the context of the formalin test, but also supports the refinement of the proposed formalin method. It should be noted that control littermates for the *Pink1* group showed a more pronounced response to the formalin in comparison to the other control groups. We trust the behaviour observed represents the natural variation it can be obtained when performing behavioural tests and therefore we can only emphasise the importance of having control littermates when performing these experiments. We hypothesise that there is not a biological obvious reason that can explain the variation observed apart from the fact that they are a different cohort of animals and thus likely to differ in experimenter, day and uncontrollable variances in their cage environment (e.g. genotype of parents). Further to the pain phenotype observed in the *Pink1* knockout line, we also screened for any distinct phenotype observed for the *Slit1* knockout mice. Despite previous studies suggesting a role for *Slit1* in neuronal development ^32, 33, 76^, our data shows that global deletion of the gene does not lead to any alteration in pain behaviour in the formalin test. Together, these results demonstrate that the refined formalin test proposed in this study can be broadly used, not only to test the efficacy of drugs, as shown with gabapentin, but also to evaluate pain phenotypes in newly generated transgenic models.

In conclusion, in this study we present a refinement to the already established formalin test. We propose that the use of an inhalable anaesthetic during the injection of formalin is not only a reliable method to improve consistency when injecting the compound, but most importantly, represents a valuable refinement. We show that this method complies with the 3Rs sought by ethical committees, as well as meeting the additional 3Rs (relevancy, robustness and repeatability) sought by scientists ^13^. Moreover, we demonstrate that the test is sensitive enough to screen for possible pain phenotypes and suggest that diminishing the first phase of the formalin test has little consequence on the global pain response of the animal.

## Data availability

### Underlying data

Figshare: Lopes *et al*., A refinement to the formalin test in mice - Extended data. https://doi.org/10.6084/m9.figshare.8230655.v3 ^38^

This project contains the following extended data:

- Data Fig_2_Males Females.csv (raw data underlying Figure 2, including each animal’s gender)
- Data Fig_3_Males Females.csv (raw data underlying Figure 3, including each animal’s gender
- Data Fig_4_Males Females.csv (raw data underlying Figure 4, including each animal’s gender
- Data_Fig_5_Males Females.csv (raw data underlying Figure 5, including each animal’s gender)
- Data_Fig_2.csv (raw data underlying Figure 2)
- Data_Fig_3.csv (raw data underlying Figure 3)
- Data_Fig_4.csv (raw data underlying Figure 4)
- Data_Fig_5.csv (raw data underlying Figure 5)

### Extended data

Figshare: Lopes *et al*., A refinement to the formalin test in mice - Extended data. https://doi.org/10.6084/m9.figshare.8230655.v3 ^38^

This project contains the following extended data:

- Supplementary Figure 1 (Graphs containing the distribution of males and females across the experiments).
- Lopes_ *et_al*.,_2019_Power_Calculations.pdf (details of power calculations for each experiment)

Data are available under the terms of the Creative Commons Attribution 4.0 International license (CC-BY 4.0).
