# Supplementary material for: A refinement to the formalin test in mice
Source: F1000Res. 2019 Aug 2;8:891. Originally published 2019 Jun 20. [Version 2] doi: 10.12688/f1000research.18338.2 (PMC6707399; doi:10.12688/f1000research.18338.2)
Supplement: Peer review history

# Peer review: Reviewer response for version 2

**DOI:** 10.5256/f1000research.22123.r52019

**Published:** 2019-08-16

**Authors:** Nora Bourbia

**Affiliations:** 1 Public Health England, Chilton, UK

Thank you for revisiting your manuscript and replying to the reviewer questions.

 It is unfortunate that you can't repeat the experiment but you do have a valid reason as well as you have given a response about the specific control group results.

 Thank you for testing sex differences and adding the information on the supplementary data. However, you should use the word sex and not gender.

I confirm that I have read this submission and believe that I have an appropriate level of expertise to confirm that it is of an acceptable scientific standard.

# Peer review: Reviewer response for version 1

**DOI:** 10.5256/f1000research.20060.r50152

**Published:** 2019-07-22

**Authors:** John Riddell, Andrew Todd

**Affiliations:** 1 Spinal Cord Group, Institute of Neuroscience and Psychology, College of Medical, Veterinary and Life Sciences, University of Glasgow, Glasgow, UK

In this article, the authors demonstrate a refinement of the widely-used formalin test. They show that if the formalin injection is performed while mice are under brief general anaesthesia (Sevoflurane) the second phase behaviour is largely unaltered, even though the first phase behaviour is largely lost. Since the second phase is the more useful measure, this means that the procedure can be performed in a less stressful way, resulting in a significant improvement in animal welfare.

 The results of the study are generally convincing, and the article is clearly written and well illustrated. One concern is the relatively high level of pain behaviour in the control group for the Pink1 study (Fig 4). While the area under the curve for other groups is typically around 1000, for the controls in Fig 4 it is nearly 2000. In fact, compared to other cohorts, the Pink1-/- mice seem to have normal behaviour, while the control group show an exaggerated response. Some explanation is needed here. Also – what are the units for area under curve?

 Minor points:

  

- It would be helpful to have some explanation of why the Pink1 and Slit1 mouse lines were chosen for this study, at the end of the Experimental Animals paragraph in the Methods section.
- Page 4: In the 4 ^th^ line of the Results section "it is" should be inserted before "being" – otherwise it would be the experimenter who received the injection.
- Page 5: left column, 6 ^th^ line "led" rather than "lead".
- Page 6: left column, 6 ^th^ line – omit "seem to".

We confirm that we have read this submission and believe that we have an appropriate level of expertise to confirm that it is of an acceptable scientific standard.

# Response

**Published:** 2019-07-30

**Authors:** Douglas Lopes

**Affiliations:** King's College London, UK

**We would like to thank the reviewers for the very positive reviews of our paper. The reviewer’s comments were very helpful, and we have now revised our manuscript to address all of the issues raised.**

*The results of the study are generally convincing, and the article is clearly written and well illustrated. One concern is the relatively high level of pain behaviour in the control group for the Pink1 study (Fig 4). While the area under the curve for other groups is typically around 1000, for the controls in Fig 4 it is nearly 2000. In fact, compared to other cohorts, the Pink1-/- mice seem to have normal behaviour, while the control group show an exaggerated response. Some explanation is needed here. * 

**This point was was also raised by the other reviewer and addressed accordingly.**

**We trust the behaviour observed represents the natural variation it can be obtained when performed behavioural tests and therefore we can only emphasise why it is important to control with animals that have been bred at the same time. Although we understand and acknowledge the reviewer’s concerns, we trust the data is a true representation of the mice behaviour for this particular line. As demonstrated on the graph (Supplementary Figure 1 B), all the control animals used in this test were above the average control response in the other figures (above ~250 sec.), we therefore believe it is unlikely the findings are a false positive. We hypothesise that there is not a biological obvious reason that can explain this variation apart from the fact that they are a different batch of animals and thus likely to differ in experimenter, day and uncontrollable variances in their cage environment (such as genotype of parents, litters, litter size, etc.). Unfortunately, this line is no longer promptly available (it has been cryopreserved and archived) and therefore we are regrettably unable to repeat these experiments to investigate this further.  We have however, added a sentence in the manuscript pointing out the differences observed and explaining it as above.**

*Also – what are the units for area under curve?*

**The AUC was calculated in relation to the pain response (sec) over time. This has been added to the materials and methods**

*Minor points:*

*It would be helpful to have some explanation of why the Pink1 and Slit1 mouse lines were chosen for this study, at the end of the Experimental Animals paragraph in the Methods section.*

**These sentences were added: The transgenic lines chosen in this study were part of a parallel neuroscience program study being carried out at the MLC. In addition, it has been suggested a link between Pink1 and nociceptive processing (48,49) and Slit1 expression and peripheral injury (50–55).**

*Page 4: In the 4th line of the Results section "it is" should be inserted before "being" – otherwise it would be the experimenter who received the injection.* **This has been amended.**

*Page 5: left column, 6th line "led" rather than "lead".* **This has been amended.**

*Page 6: left column, 6th line – omit "seem to".* **This has been amended.**

# Peer review: Reviewer response for version 1

**DOI:** 10.5256/f1000research.20060.r50153

**Published:** 2019-06-26

**Authors:** Nora Bourbia

**Affiliations:** 1 Public Health England, Chilton, UK

Douglas M Lopes et al. have addressed the question of whether it is possible to refine the formalin test without affecting the behavioural outcome important in pain research. They have shown that the use of anaesthesia (sevoflurane) at the injection time reduces the distress of the handling and formalin injection, and the behaviour associated to the first phase while conserving the behaviour associated to the late phase of the formalin injection test.

 The experiment and the method are well performed, clear to follow for reproducibility and well designed to answer to the initial question.

 The study mentioned that both female and male mice were used which is very valuable and important in research. However, and except if I have missed it, I am not aware of the presence of a statistical test performed to confirm the absence of a sex-difference in behavioural responses to the formalin test in this study because sex difference can be a factor influencing the pain perception. A clarification would be appreciated as well as the presence of the sex in the source data.

 Additionally, it is mentioned that wildtype C57BL/6 mice were used for the gabapentin and sevoflurane experiments but without mentioning the exact background while it is clearly mentioned for the transgenic mouse study (C57BL/6NTac). Pain-like behaviour can be influenced by the mouse strains, also behavioural differences have been observed between C57BL/6N and C57BL/6J mice. It would be valuable to mention the exact background for a full reproducibility.

 The results of the sevoflurane effect, the gabapentin treatment, and Slit1 KO studies are very clear. I did, however, notice a discrepancy in the data from the control group of the Pink1-/- study compared to the control group in Slit-/- study (both C57BL/6Ntac background) and the wildtype C57BL/6 mice in the anaesthesia/gabapentin experiment. The average pain behaviour in the control mice of the Pink1-/- study is almost twice higher than all other group controls and the Pink1-/- mice between the time 20 and 35 min. Could the authors provide a possible explanation about this discrepancy? This might lead to a potential false positive result of Pink1 effect in this study. Ideally, to confirm the effect of Pink1-/- on the behaviour induced by the formalin test, it would be appreciated to repeat this test (Pink1-/- vs its littermate control) with again randomisation and blinding.

I confirm that I have read this submission and believe that I have an appropriate level of expertise to confirm that it is of an acceptable scientific standard, however I have significant reservations, as outlined above.

# Response

**Published:** 2019-07-30

**Authors:** Douglas Lopes

**Affiliations:** King's College London, UK

**We would like to thank the reviewers for the very positive reviews of our paper. The reviewer’s comments were very helpful, and we have now revised our manuscript to address all of the issues raised.**

*The study mentioned that both female and male mice were used which is very valuable and important in research. However, and except if I have missed it, I am not aware of the presence of a statistical test performed to confirm the absence of a sex-difference in behavioural responses to the formalin test in this study because sex difference can be a factor influencing the pain perception. A clarification would be appreciated as well as the presence of the sex in the source data.* Thank you for pointing that out. **We have now uploaded the files containing the required information. We have also conducted statistical tests comparing males and females across the different experiments (except for the experiments using gabapentin, where only males were used). Furthermore, we uploaded an additional figure where the graphs contain each individual animal (females in red circle and males in blue square). The figure legend contains the details for each one of the tests. Both the visual representations and statistical analysis demonstrate no differences between the genders. A sentence stating the above findings and link to the Supplementary Figure has been added to the Material and methods section (Experimental Animals).**

**It should be noted that although we performed the appropriate statistical test to compare sex-differences and observe no differences, for some of the groups analysed the outcome might be pursued as not ‘scientifically meaningful’, as the numbers of animals in each subgroup were below the ‘n’ number recommended by some recent published guidelines (Curtis, et al., 2018 Br J Pharmacol. Apr; 175(7): 987–993).**

*Additionally, it is mentioned that wildtype C57BL/6 mice were used for the gabapentin and sevoflurane experiments but without mentioning the exact background while it is clearly mentioned for the transgenic mouse study (C57BL/6NTac). Pain-like behaviour can be influenced by the mouse strains, also behavioural differences have been observed between C57BL/6N and C57BL/6J mice. It would be valuable to mention the exact background for a full reproducibility.* ****The background has now been added (C57BL/6NTac).**

*The results of the sevoflurane effect, the gabapentin treatment, and Slit1 KO studies are very clear. I did, however, notice a discrepancy in the data from the control group of the Pink1-/- study compared to the control group in Slit-/- study (both C57BL/6Ntac background) and the wildtype C57BL/6 mice in the anaesthesia/gabapentin experiment. The average pain behaviour in the control mice of the Pink1-/- study is almost twice higher than all other group controls and the Pink1-/- mice between the time 20 and 35 min. Could the authors provide a possible explanation about this discrepancy? This might lead to a potential false positive result of Pink1 effect in this study. Ideally, to confirm the effect of Pink1-/- on the behaviour induced by the formalin test, it would be appreciated to repeat this test (Pink1-/- vs its littermate control) with again randomisation and blinding.*

**Thank you for pointing this out. We trust the behaviour observed represents the natural variation it can be obtained when performing behavioural tests and therefore we can only emphasise why it is important to control with animals that have been bred at the same time. Although we understand and acknowledge the reviewer’s concerns, we trust the data is a true representation of the mice behaviour for this particular line. As demonstrated on the graph (Supplementary Figure 1 B), all the control animals used in this test were above the average control response in the other figures (above ~250 sec.), we therefore believe it is unlikely the findings are a false positive. We hypothesise that there is not a biological obvious reason that can explain this variation apart from the fact that they are a different batch of animals and thus likely to differ in experimenter, day and uncontrollable variances in their cage environment (such as genotype of parents, litters, litter size, etc.). Unfortunately, this line is no longer promptly available (it has been cryopreserved and archived) and therefore we are regrettably unable to repeat these experiments to investigate this further.  We have however, added a sentence in the manuscript pointing out the differences observed and explaining it as above.**
